# Lecture Introductory to the Course on Materia Medica and Hygiene, Delivered before the Medical Class of the University of Buffalo, Nov. 28th, 1869

**Published:** 1869-12

**Authors:** Charles A. Lee


BUFFALO

### Medical and Surgical Journal.

VOL. IX. DECEMBER, 1869. No. 5.

### Original Communications.

ART. I.—*Lecture Introductory to the Course on Materia Medica and Hygiene, delivered before the Medical Class of the University of Buffalo, Nov. 28th,* 1869. BY CHARLES A. LEE, M. D.

Published by request of the Class.

The study of Materia Medica is designed to acquaint the medical student with the uses and powers of remedies, and prepare him to make a proper selection to meet the ever-varying phases of disease. The importance of this kind of knowledge cannot be appreciated until the actual emergencies of practice arise, and the necessity becomes apparent of an extended and thorough knowledge of the weapons for combating disease.

As Anatomy, Physiology and Chemistry form the basis of all scientific medicine, so they form the foundation of Therapeutics—without them, all other knowledge is empirical and unsatisfactory. But it is the certainty and excellence of our diagnosis which gives to therapeutical medicine such an advantage over that of antiquity. We are no longer obliged to grope our way in the dark, and guess at the causes, nature or seat of, disease; a knowledge of these enables

NOTE.—This lecture was written with no view to publication, nor does it lay any claim to originality. The ideas and, in some cases, the language of others has been freely used. But, as the class has asked for its publication, the writer has reluctantly consented. L.

us to aim directly at its removal or alleviation. By the aid of chemistry, the materia medica has been vastly simplified; for, instead of the crude articles of bulky and distasteful drugs, we now accomplish vastly more by using their concentrated active principles. Every year this branch of medicine is becoming more and more simplified, and more certain and efficient. Useless and inert remedies are dropped from our list, while others of greater power are added. Pharmaceutical Art, the handmaid of Practical Medicine, walks hand in hand with Therapeutics, while Physiology and Pathology remove all obstacles from their path. It is difficult to understand how physicians of a former age could place much confidence in the power of drugs to cure disease, as long as they were ignorant of its conditions, its causes, nature and seat. It has required the experience of many centuries to teach us what we know of remedies, or the natural history of diseases—that is, the course they will run uninfluenced by medication; and there is a large and popular school of practitioners among us, that seem not yet to have learned even this, but ever reasoning on the *post hoc, ergo propter hoc* principle, attribute all recoveries to the specific antidotal power of drugs, giving nature, with her conservative and recuperative forces, no credit whatever. But the practitioner who knows so little of pathology as to be unable to discriminate diseases, so little of physiology as to confound morbid with healthy function, and so little of signs and symptoms as to be unable to determine their value and significance, has mistaken his calling, and would do better to follow some mechanical pursuit which requires little or no thought, no reasoning faculties and no logic.

I doubt not, gentlemen, that some of you come to the study of the materia medica with more or less of prejudice and preconceived bias. You may have unconsciously imbibed some of the scepticism which characterizes the age, in regard to the curative powers of drugs, or the value of specific medicine. It were strange, indeed, if you had not; an age of credulity is apt to be followed by one of scepticism. It is so in religion, and it is so in medicine. You have heard that, with the varying views of physiology, pathology and medical philosophy, the department of therapeutics—the only part of medicine for which the public cares—has, of late years, lost its stable foundations, and is of no more account. Slight observation must have revealed to you the fact, that a fashionable and widely prevalent doctrine is, that drugs are of little or no service in the cure of disease; that venesection should be wholly dropped; that counter irritation is barbarous; that emetics are savage—a relic of canine and feline practice; cathartics abominable; and that our true stronghold is the "efforts of nature." Expectantism and nihilism are the pass-words of the hour. You have, perhaps, even heard some physicians say, "I don't believe much in drugs;" and they may add, "formerly I used to have twenty remedies for every disease, but now I have twenty diseases for one remedy." Such practitioners have dropped blood-letting long ago; not because it was not often very useful, but because the various phases and hordes of charlatanry have proved too strong for them. It is, certainly, a most remarkable fact, that after this remedy has been successfully used by physicians for many centuries, it is now scarcely mentioned, either in our schools or standard works on the practice of medicine; and the great mass of our physicians rarely, if ever, use a lancet, or even carry one in their pockets. The profession yields to popular clamor, science to sciolism, and "*medicine expectante*" is the result. Fortunately, so far as bleeding is concerned, other remedies have been introduced which frequently answer as a substitute, such as aconite, veratrum and chloroform.

It will be my aim, in the present course of lectures, to vindicate the claims of drugs as efficient agents in the cure of disease; to show that we are not only the interpreters of nature, but her aiders and controllers; and that, by means of the instruments furnished us by the vegetable, animal and mineral kingdoms; and that, if we do not employ them, we are more stupid than the lower animals, whom instinct prompts to resort to them for the relief of their sufferings. An adequate knowledge of the pathology of disease will go far to limit your endeavors to what is rational and practically attainable; for it is only the half educated physician who promises impossible cures. Such knowledge will teach you what is feasible in our art, and what is not, so that you may not attempt what is impracticable, or be disappointed because you cannot always accomplish your aims. Age, knowledge and experience, it is true, tend to narrow the scope of our ambition as well as our expectations. In this way, chiefly, age and experience may, and do, tend to lessen the amount of drugs administered; for, when we have learned, by long and careful trials, what medicines can or cannot do, we are prepared to spare our patients the trouble or the danger of hypermedication. The allegation is a very old, and probably a true one, that young practitioners expect too much from drugs, and, at the same time, undervalue the importance of hygienic agents, which, if important to preserve, are equally so to restore health when lost. It is very certain that these latter always rise in value in proportion to the extent of our experience in the treatment of the sick; and because this is so, physicians, as they advance in age, are supposed to undervalue the efficacy of medicines proper. But this charge is quite unfounded in regard to all well educated practitioners who keep abreast with the science of the times, and thoroughly posted in therapeutical knowledge. Those who grow old without study, who see but never observe or reflect, may, and often do, salve over their deficiencies, by avowing their scepticism as to the utility of drugs and medication; but, when we hear such comments on the inefficiency of our therapeutical efforts, we may at once draw the conclusion, that the utterer of such wisdom is merely finding some excuse or cover for his own ignorance and short-comings.

But, gentlemen, whatever may be the scepticism of some physicians in regard to the value of medical treatment, our warfare against disease is not a vain warfare. Since the days of Sydenham and Bacon, at least, every year has been marked by progress in our science; at some periods it has been slower than others, according as the inductive or deductive philosophy has prevailed, but the advance has been constant. It is true the elements with which we have to deal in our therapeutical and biological studies are numerous and diversified, subject to an almost infinite variety of combinations; but, still they are governed by laws, not caprice—laws ascertainable by study and observation—a knowledge of which lies at the foundation of all progress, and which should constantly stimulate to further and more determined efforts. To arrive at rational and stable conclusions in therapeutics, we must appreciate the correlation of all ascertained facts in the several departments of science; of coordination, by careful comparison of all the materials acquired by observation and rigid induction. We need, it is true, a more precise and accurate acquaintance with the specific powers and virtues of drugs—not by experiments on healthy individuals, for they can do nothing when nothing is to be done; but, by careful observations and experiments made in disease as well as health. Who would, for example, ever have learned that opium would relieve pain or spasm, if it had not been given for their relief, and when they were present, and so of all other drugs. Let us not undervalue the discoveries of the past. Look at the inestimable value of our materia medica. What admirable remedies for allaying pain and spasm, and producing sleep; of benumbing the sensibility of nerves so that the knife of the surgeon is no longer felt; of extinguishing fevers; annulling inflammation; putting a stop to the paroxysms of intermittents; curing or preventing epileptic attacks; controlling the progress of gout and rheumatism; correcting morbid states of the blood, its secretions and excretions; of neutralizing and destroying poisons, whether introduced by design or accident; of checking the progress of tuberculosis, and curing scorbutic and scrofulous affections; in short, of alleviating or curing a large proportion of the maladies which afflict the human family.

When you hear it asserted that the philosophical spirit of the age is opposed to a belief in medicines, you must recollect there is a fashion in medicine as well as in every thing else. But there are signs of a coming reaction, going to show that, in the practice of our profession, drugs are again to find their legitimate place; that blood letting, local and general, is again to occupy its proper sphere. We are now living in a transition period of medicine, when the elements of our science, and even its foundations, seem to be undergoing re-arrangement. Our former notions of structure and function, and vital changes, were, doubtless, partial to some extent, lacking in precision, and often incorrect; and so, also, were some of our notions in regard to the physiological and curative powers of drugs and their mode of operation. Within my own recollection the pathology of many most important and fatal diseases has undergone a complete change, and a corresponding change of treatment has followed. I need only refer to tubercular diseases, which formerly were supposed to have an inflammatory origin, and treated with antimony, bleeding, and counter-irritation, but are now attributed to morbid changes in the blood, due to faulty, primary and secondary assimilation of food, and often controlled or cured by appropriate stimulants, tonics, and nutriment; and so of many other affections. We have at length reached the grand generalization, that a large proportion of local disorders and organic affections have a constitutional origin, and that we must look primarily to the assimilating functions; the healthy blood-making processes, which imply sound innervation; the due activity of the secretory and excretory functions; proper vitalization of many constituents, as lying at the very foundation of health, as well as the successful treatment of deviations from it. We have sought to individualize disease too much, to seek specifics for every malady known to medical nomenclature instead of adapting our remedies to the correction of vital actions, and controlling physiological conditions by proper hygienic agents, as well as wisely selected medicines.

He must indeed be blind, as well as sceptical, who denies the existence of certain special powers in drugs—such as iron to remedy a deficiency of globuline in the blood, or of fish oils to nourish the fatty tissues by furnishing a richer chyme, &c. Who can doubt that we can, by the use of appropriate remedies, render old deposits of inactive character more susceptible of organization, and therefore of removal; that we can hasten the absorption of the plastic exudations of inflammation; that we can restrain excessive secretion and excretion, and so prevent waste; that we can strengthen the nervous and muscular system by suitable tonics and stimulants; or, that we can lower morbid susceptibility to reflex impressions by proper sedatives; in short, that we can alleviate or cure a vast proportion of human maladies?

Those who distrust the value of medication refer us, with an air of triumph, to the acknowledged fact, that many diseases are self-limited, and run their course with safety without the aid of medical treatment. Admitting this, it by no means follows, that their violence may not be abated, and their tendencies to permanent evil be prevented by proper management. There is no disease, however trifling in appearance, but which requires watching by a careful physician: none in which serious complications may not suddenly occur requiring medical interference. Many of our incidental diseases may be arrested in their progress by appropriate treatment, while many of our fevers, when no complications arise, need no other remedies than pure air and suitable nourishment.

Gentlemen, while some eminent practitioners hold that therapeutics is wholly an empirical science, if, indeed, a science at all, I must maintain that it derives most important aid from physiology and pathology; and, in fact, that these have led to the discovery of some of our most important remedies; and that experiments on the lower animals, and on healthy human beings, have been of great scientific value. While we reject no conclusions derived from carefully observed and well ascertained empirical facts, we must not reject those founded on the physiological action of drugs. It is by combining the two methods, that therapeusis has made more progress during the last quarter of a century than for the preceding one thousand years. Look, for example, at the successful treatment of epileptic and paralytic affections by Dr. Brown-Sequard, based wholly on his physiological experiments and pathological facts—results which would never have been attained by relying on empirical observation. You can judge as to the value of such knowledge when you look back and find the recipes of Galen, sometimes containing a hundred different articles, and most of them inert, followed, without variation, for more than one thousand five hundred years. When you see healing medicaments, so called, composed of oil and wine, and a thousand inert or irritating substances, used for the healing of wounds, ulcers and sores; and when you find warm drinks, and the heating regimen, prescribed for fevers, inflammations, small pox, and the whole class of exanthematous affections, without a single doubt of their efficacy. To establish therapeutics on a firm foundation, and arrive at trustworthy and useful rules for the treatment of disease, while we need the most careful and intelligent investigation then of the curative effects of medicines, we must also keep pace with physical diagnosis, physiology, pathological anatomy and pathological chemistry.

Nor should we, through fear of the evils of perturbation, adopt the error of postponing all medical treatment of disease until our knowledge of the action of medicines, and our insight into pathological processes, be so far advanced that our means of cure be self-evident. Such a goal can never be reached, and in the inscrutable designs of Providence, it may never have been designed to be attained; for it is idle to hope that the time will ever come when a medical prescription should be the simple resultant, as Niemeyer expresses it, of a computation of known quantities.

Gentlemen, if therapeusis is to derive no aid from the other departments of medical science, and it must be studied by itself as an independent and peculiar branch of knowledge; if the empirical method of investigation is the only rational and proper one for the study of therapeutics, we may abandon the hope of any essential progress for many centuries to come.

I would fain hope that you come to the study of the materia medica in a liberal catholic spirit, with no blind attachment to any system or doctrine, whatever. Humility becomes us all, and especially those just entering on the threshold of medical study, who cannot be supposed qualified to sit in judgment on the merits of medical systems, or the advocates of any fashionable school or theory. It is your duty to study carefully, with all the best aids within your reach, the virtues, properties and uses of every weapon in the armory of the materia medica, no matter what its *modus operandi* may be. The only question should be, to what diseased conditions is it suited; and it matters not, so far as the suffering patient is concerned, whether the medicine is believed to act homœopathically or allopathically, provided it only act beneficially; and whether it act in the one way or the other, is probably known only to Him who formed us what we are. Why should a sane physician spend his time in trifles—in splitting hairs as to the mode of operation of remedies; in investigating the bearings of a so-called *law*, (*similia similibus*,) whose very existence is doubted; the value of a mode of practice, which has been said to be, "not any thing, so much as a nothing, which looks like something." Why place our trust in mythic potencies, or infinitesimal divisions of a dose, when we know we are not endowed with any such intense susceptibility to impressions, the existence of which would be imcompatible with the continuance of human life for a single day. How any sane mind, witnessing the value of medicinal agents which have the acknowledged power of arresting and curing disease, should dare to rely on a species of therapeutic nihilism, I know not; and especially when the age is replete with discoveries tending to enhance the value and the certainty of our art; either by improving our knowledge of disease, or by assisting our comprehension of the action and virtues of remedies. We are every day accumulating sure and authentic therapeutic facts unknown to our predecessors: every system yields some valuable fruit to scientific and practical medicine; and it becomes us not to enrol ourselves under the banner of any party leader, or clog our steps with the fetters of any pretended reformer. True science repudiates any such course; catholicity scorns all such slavery. When you know that the smallest well authenticated fact in therapeutics is of profound importance, you will, I trust, be inspired to persevering researches and study requisite to raise our art to the rank of an exact science—one which may not only take equal rank with the other departments of medicine, but be acknowledged as standing at their head.

However medicines may have been regarded in the past, there can be no rational doubt that they have accomplished vastly more good than evil. The evils of hypermedication, and perturbative treatment, are necessarily incidental to the administration of any active remedies, unless human judgment be infallible, which is not claimed. Evil is ever mixed with good. It is the dispensation of an all-wise Creator. The same intelligence has implanted in the human heart an instinctive belief in medication; and there has been no nation, barbarous or civilized, where this faith in the healing art has not existed. With Prof. S. H. Dickson, I would say, "far be it from me to defend, or even excuse, a promiscuous or indifferent employment of agents, possessed of whatever powers. It is the very essence of our art, the very purpose and object of our science to distinguish and appreciate the circumstances and occasions for our interposition; to enjoin abstinence from all blind and indeterminate action; to enforce the restraints of prudence and ensure the guidance of reason and experience. But I will not shrink from saying that reason as well as instinct, and the highest prudence, seem to me to justify, or rather to demand, an unhesitating interference—even if simply tentative—where our knowledge and experience fail us, in preference to an inactive, stolid submission. We must examine such contingencies on every side, and surrender ourselves to the dictation of some hopeful analogy, or some plausible, even if conjectural, *rationale*. It is plain, indeed, that without this instinctive, nay, we may call it involuntary, effort to cry for help, there never would have originated an art of healing, never existed a divine science of medicine, such as we are hopefully engaged in building up. As a universal rule, in the obscurest ignorance, the most misty doubt, I would maintain that perturbation is better than inaction: in the former there is hope, even if we incur unavoidable danger; in the latter, nothing but black despair—unpardonable acquiescence in suffering and injury."*

It is no valid objection to our remedies that they have power; indeed, if they had not, they would do neither good nor harm. What is needed is, to know how to use them prudently, safely and usefully; and this is the object of a course of instruction in this branch. First, to avoid all harm; secondly, to do all possible good by their use.

Passive treatment may answer in passive diseases; in those of an active character, something more is needed. A system of practice which can do no harm if it do no good, is a nonentity. Ours is both an art and a science; its principles, rules and reasons, so far as I am acquainted with them, I shall endeavor to impart—though, so far as it is an art, of course, much of it is not teachable. The faculty of judgment is incommunicable, and medical sagacity has been called transcendental, as extending beyond the combination of all that can be taught by precept.

I have expressed a belief that a reaction is now going on in regard to bleeding, and other active modes of treatment; and this opinion is based on what I hear and see around me. Physicians in extensive practice, who have not unsheathed their lancets for a quarter of a century, are again resorting to their use; leeches are again in demand; cupping is not an unfrequent operation; and the belief is a very prevalent one, that the physician has something more to do than to wait and "expect." I am old enough to have witnessed a complete medical cycle. I can remember when promiscuous blood-letting was the order of the day; when mercury and antimony were regarded as the sheet-anchor in most fevers and inflammations. Diseases were then unquestionably of an acute and asthenic type, and needed depletion. There was no difference of opinion on this point. Then, gradually, dating from about the year 1832, when the cholera first appeared in this country, it is generally con-

*Studies of Pathology and Therapeutics, p. 176.

ceded, there slowly came on a change in the character of disease, as well as the constitutions of the subjects; both became asthenic or adynamic, and stimulants and tonics took the place of depletion and sedatives. The present generation of physicians, who have come upon the stage since then, and who have not witnessed this change of type and diathesis in its fullest extent, may possibly doubt its existence, or attribute a change of treatment to the influence of homœopathy, or some other system. But this would be a great mistake. The best observers, the aged practitioners, whose experience dates back nearly half a century, will tell you that this asthenic constitution of disease, and of subjects, culminated some time ago, and is now passing, or has, in a measure, passed away. There is much truth in the remark of Prof. Dickson, when he says: "I do not know—I scarcely suppose—that cathartics are more demanded; but I am satisfied that depletory measures of every other character, venesection especially among them, are not only coming again more into fashion, but are really more frequently called for and better adapted to the general requirements of ordinary practice." This able writer and teacher very appropriately refers to the experience of our late war in proof of his position; during which he says, "might have been seen under the most depressing contingencies of imperfect nutrition, shelter, ventilation, and clothing, the loss of blood to be far less impressive for evil than would have been supposed."

The stimulating practice, so excessive and so rife for many years, and especially during the war, in army practice, has been gradually yielding to more rational methods; and even writers like Prof. Bennett, of Edinburgh, who, a few years since, advised scarcely any treatment except stimulants for almost every disease, now recommends "a little careful depletion," now and then "leeches and cupping," laxatives, emetics, blisters, quinine, iodide of potassa, cod-liver oil, &c.

The successful practice of medicine is nothing more nor less than the application of common sense, in the use of remedies, with whose virtues and powers the practitioner has made himself acquainted. The rational physician ever follows the promptings of nature. If he finds a patient troubled with anorexia, he abstracts solid food; if there be heat and thirst, he prescribes ice, cool, effervescent or acidulated drinks internally, with cold or tepid sponging externally; if prostration and languor oppress the patient, he recommends quiet and rest; for the head-ache and delirium of an overtasked brain, he advises relaxation and abstinence from study, with perfect repose of body and mind; if light and noise annoy and disturb, silence and a darkened room bring relief; but if the great *Hahnnemanic law* of cure be the guiding principle, let the practitioner immediately aim to add somewhat to these morbid conditions by intensifying their causes, and the cure is accomplished. If common sense revolt, and reason rebel, then so much the worse for both.

We have learned to rely on the specific antidotal power of *quinine* in the destruction of malarial poison, and the arrest and cure of the whole class of fevers depending on it. Why, then, should we despair of finding other specifics for other specific poisons, and diseases proceeding therefrom; our endemic, epidemic, pestilential and malignant maladies, which are now more than decimating the race? We have an effectual antidote to small pox in the vaccine disease; can we doubt there are, also, antidotes to all other exanthematous poisons—that of measles, scarlet fever, rubeola, typhoid fever; as well as of typhus, cancer, asiatic cholera, diptheria, &c.? Specific poisons, undoubtedly, have their specific antidotes; which remain to be discovered by future research. There is some reason to hope that we already have such a general antidote in the alkaline and earthy *sulphites*. Though known in medicine for more than forty years, and employed successfully by M. Chaussier, in Paris, twenty-five years ago, they remained almost unknown to the profession till Prof. Polli, of Milan, again brought them into notice some ten years since. From that time till the present, they have been more or less employed, until their effects in general, and the safety of their administration are now widely known, and their value confirmed by the testimony of many eminent men in various parts of our country as well as Europe. They have not only been successfully used for the cure of intermittent, remittent and typhus fevers, but also of small pox, measles, erysipelas, cerebro-spinal meningitis, scarlatina, yellow fever, puerperal metritis, and indeed most diseases depending on blood poisoning. That these sulphites are absorbed into the blood, and therein exert their antiseptic properties directly upon the materies morbi, which gives rise to the disorders, is generally conceded by those who have studied their operation most carefully.

I have already alluded to the immense advantages our science has derived from the more accurate diagnosis which distinguishes our age from every preceding one, and need only refer, in addition, to physical exploration in diseases of the chest—the ophthalmoscope in those of the eye—the laryngoscope of those of the larynx—and the sphymograph, which aids so essentially in determining the character of the pulse in diseases generally. I am reluctantly compelled to pass over the many admirable and new remedies which have been successfully introduced into practice in our times, including our indigenous plants and their active principles, alkaloids, resinoids and neutrals; but I may, in passing, refer to that sovereign remedy for asthma and other affections, the respiration of compressed air, so highly appreciated by the faculty in Germany and France, and so justly extolled by Niemeyer in his recent work on Practical Medicine, (vol. 1, p. 89.)

In the present strife and competition for medical business, the question has come to be, not so much what is best for the patient but what is safest for the practitioner. What course of treatment will give most satisfaction to the family and friends in case of the patient's death? Bleeding, against which so much unjust prejudice has been raised by quacks and their abettors, must at all events be avoided, else it may be said, the patient died from the treatment, and not from the disease. The bug-bear of weakness is always a ready excuse for the timid and routine practitioner, who hesitates not to let his patient perish by neglect of the only remedy that promised success. No matter; he saves his own reputation, so far, that it cannot be said he caused the death of his patients by his active treatment; he only lets them die without any treatment at all!

Time would fail me to speak of the vast additions to our therapeutic knowledge during the last half century; greater, I doubt not, than during all the centuries since the christian era. And such are the present indications of the future usefulness and glory of the divine Art of Healing, that we are fully justified in indulging the most sanguine anticipations of still more rapid progress in the future.

The discovery of the prophylactic power of vaccine virus; of quinia and the other vegetable alkaloids; of anæsthetic agents; of hypodermic injections in neuralgia and other painful affections; of the process of atomization of concentrated remedies for internal as well as external application; these, and many other new remedies and processes, each for itself, would serve to form an important epoch in the history of our science. Owing to the discoveries and improvements in medicine, the value of human life has been already nearly doubled, and the present cultivation of physiological and sanitary science, promises the most important benefits and results, as connected with the health of individuals and communities.

In the study of this department of medicine, gentlemen, you are not to forget that the sciences, and their various branches, are all bound together by indissoluble links. We separate them for our convenience; but, when we analyze all the facts and phenomena of the external world, there would seem to be, underlying all, one *great force*—one primal mover, out of which, with unvarying precision, the great phenomena of the universe proceed. To this result all recent physical researches seem to tend. *Heat*, itself, seems but a mode of motion; heat, at certain temperatures, becomes *light—light*, in its turn, as we see in the photographic art, becomes the source of *chemical change*—chemical force becomes *galvanism*—galvanism is converted into *magnetism—motion* generates *electricity* and *heat*—galvanism and magnetism produce *heat, light, electricity* and *motion.* We may even go a step further. It is the heat and light which emanate from the sun, which causes plants to live. The heat and light become chemical force in the plant, all growth becomes active, the phenomena of vitality are manifested. Suppose the rays of the sun are withdrawn—the phenomena of life diminish; chemical change ceases—plant life grows sluggish—the plants drop their leaves—some die—and a dormant vegetation characterizes our winter months—to be roused into life and activity again when the solar warmth of spring returns.

You see here what is called a correlation of physical and vital forces; but this correlation does not end here. During the life of the plant, there has been deposited sugar and starch, and gluten, in its roots, in its stem, its leaves, and its seeds, These are the food of animals. Withdraw this food and they die. The chemistry of the sun's rays has created these proximate principles, so called. This chemistry is undone, so to say, in the animal—it becomes animal heat—it becomes animal motion, or rather muscular force in the muscles; and that most mysterious and refined of all motions, nerve force, by which the spiritual consciousness of man becomes cognizant of sensations—accumulates ideas, reasons and becomes an intelligent, conscious and responsible being.

In this way, we can get a glimpse of a real connection of the sciences—of a dependence of one upon the other. You see that there is no sharply defined line between the various departments of science any more than there is between the different branches of medicine: each contemplates facts and principles, which are more or less common to all. Our consiousness of health and sickness is one of the most common facts of our existence; and we have no records of communities of men who are not cognizant of disease as opposed to health; and, as already remarked, who do not employ some measures, whether efficacious or not, for the cure or prevention of disease.

The subject of Hygiene will occupy some of our attention during the present session. Hygiene, as you are well aware, differs from medicine—the first prevents, the latter cures, disease. Perhaps it might properly be called preventive medicine. It is very closely connected with physiology, which teaches us the laws of health, or the laws of life; and also with chemistry, which reveals to us the nature of poisons, whether taken in with the air we breathe, the food we eat, or the fluids we imbibe. Hygiene aims to discover the causes of disease and death, and the means of so averting and altering these causes as to prevent those calamities; and, to do this, it classifies the great factors of life under *air*, *water*, *food* and *heat*, and all the various questions that hygienic enquiries bring up, may be classified under one or more of these.

Thus, there is no animal life without *air*. The humblest monad needs for its existence a supply of oxygen gas. Its life motions are derived from the oxygen producing chemical changes in its interior, and so of all other living beings. Man is but an aggregation of monads. Each living cell of which his body is made up, contributes to the aggregate of his life, only as it is acted on by the oxygen of the air. This fact lies at the foundation of a thousand hygienic enquiries and sanitary facts. It is the necessity for the oxygenation of our tissues, that gives all their importance to our enquiries into the ventilation of dwellings and work shops, of school houses, churches, stables—in fact of all places where living, breathing beings have to live. It is this fact which lies at the foundation of all our anxiety about over-crowding of tenement houses, factories and shops. By the aid of this great primal fact, we explain the unnecessary amount of disease and death from scrofula and consumption; and the more this great fact is heeded and recognized, in that proportion will longevity be promoted, and the health of cities and communities enhanced. But we are also to remember, that the air we breathe not only supplies us with oxygen, but it is the great repository of all that is exhaled from the earth, and from decaying matters on the surface of the earth, and that it often comes to us, as well as the lower animals, loaded with poisons—chemical agents which, absorbed into the blood through the lungs, work their destructive action on the frame, and either damage the functions of life or destroy existence altogether, In fact, all the great questions of endemic, epidemic, miasmatic, and contagious diseases, find their appropriate place in our enquiries into the nature of impure and poisoned air!

*Water* is another factor of organic life. Without water no chemical or vital change can take place in the living body. Water enters into the composition of all organic beings. A large number of animals have their existence determined by water. A man weighing 150 lbs. contains 111 lbs. of water in his tissues. The oxygen that vitalizes his tissues is conveyed by water. The starch, the fat, the albumen, so necessary to the existence of animals, are all digested, absorbed, and conveyed to the tissues by water. These substances, through whose chemical change life is possible, are decomposed in the presence of water, and the products of this decomposition are carried off by the agency of water. All the higher animals drink water for this very purpose; and the adult human being, on an average, in one form or another, takes from 70 to 80 ounces of water daily. Water is the most potent of chemical agents; its solvent power is equal to that of the mineral acids, and it associates itself in nature with a vast variety of compounds with which it comes into contact in the external world. It dissolves both organic and inorganic matters, hence it may become so contaminated as to be unfitted for the purposes of life From the inorganic world, it may take up the salts of lime, iron, lead, copper, arsenic and other compounds, in such quantities that, when taken into the human body, it is not only unfit for healthy life, but it may become the source of immediate disease or death. Like the air, it may become the medium of introducing those definite organic poisons, which, kindling similar poisons in the living system, are at once the source of disease to others, and the death of the individual suffering from their action. Hence, among hygienic enquiries, none, perhaps, are more interesting and important than those relating to the quality of the water we drink; and not only this, but as connected with washing, cooking and manufacturing purposes.

But something more than pure air and water is necessary for the growth and well-being of the animal organism; it requires varied compounds of carbon, hydrogen, oxygen and nitrogen, in the shape of *food*. It is very evident that the purest air and water will be no protection from disease and death, unless the human system is supplied in its food with the elements necessary for the play of those chemical forces which result in life or vital phenomena. Not only must there be food supplying the materials of combustion and nutrition, but each tissue is built up and constituted in its own peculiar way. The blood must be supplied with chloride of sodium and iron—the bones with phosphate, carbonate and fluate of lime—the muscles with potash—the bile with sulphur—the saliva with cyanogen—the nervous structure with phosphorus—the hair, teeth and nails with silica—and a diet deficient in these materials may be a source of disease, as scorbutus. Formerly the navies of the world were decimated for want of fresh vegetables. Armies have been virtually starved on an excessive diet of salt beef. Children have been sacrificed by thousands by confining them to starchy food—arrow root and corn-starch. No matter how much pure air and water are furnished, the body must have all the elements, and all the minerals, which enter into its composition, in order to ensure health. This furnishes a clue to the question, what should constitute the food of man? what is a healthy diet? Here science and instinct tend to the same goal; they reach the same results, both in the case of man and the lower animals. And, in this connection, comes up the question of nervous stimulants—of alcohol, tea, coffee, tobacco, opium, indian hemp, &c., and especially the influence of alcoholic drinks. Are they, in any proper sense, food? Do they retard the decomposition of the issues? Are they ever necessary, except as medicines? All these questions will be fully considered hereafter.

I have mentioned *Heat* as one of the factors of life. Provision for the artificial maintenance of heat is one of the proofs and signs of civilization. The naked savage may live on air, food and water. The civilized man must have warmth. There is no life where the temperature never rises above $32_o$ F.; and a little above this we find only plants and animals of the lowest types and feeblest vital powers. But as we ascend the animal scale to birds and mammalia, we find animals constructed to maintain their own temperature, and thus become independent of external sources of heat. The commonly received theory of the function of calorification in animals is, that heat is maintained by the combination of the carbon of the food with oxygen, for we see animals living in cold climates maintaining their own temperature by large supplies of food. Indeed, their whole existence seems to be thus spent—for instinct teaches them that, without food, they speedily perish from cold. And such has been the experience of the arctic navigator. So man, if his food is scanty, heaps on clothing. If his dwelling is well warmed he needs less food and clothing. The cold of our northern winters is a great enemy of life, especially to the very young or the aged —a very cold day is the death-knell of thousands. Statistics show that the greatest mortality among those over sixty occurs in the coldest weather—and this, especially, among the lower and poorer classes, who are unable to procure necessary clothing or fuel. Philanthropists ought to turn their attention in this direction, and see, if means cannot be devised to save the lives which are now destroyed by cold. This is especially necessary in such a hyperborean climate as ours. Chest affections in winter are the representatives of bowel affections in summer. But then the latter are more amenable to treatment than the former. Pulmonary affections are the scourge of our winter months.

Gentlemen, let me recommend to you to study the laws of heat in relation to the life of man, so that you will, hereafter, not only be able to direct in the construction and warming of your own houses, and arrange your clothing so as to secure to yourselves immunity from temperatures destructive of health and life, but also, to aid by your counsel and advice, so as to secure the same blessings to your friends and acquaintance, and to the community generally, where you may select your residence.

The future progress of medical science, as it seems to me, must rest very greatly on experimental and original research, which have a direct bearing on the treatment and cure of disease, and these experiments, to be worth anything, as a means of medical research, should be blended with, as well as constitute a true foundation of experience. It seems to me, gentlemen, we have now no other mode of progress before us. The history of medicine has all been written; the classification of diseases is well advanced; the symptoms of special diseases have been so fully described as to render diagnosis a certain science; the ordinary or descriptive anatomy of the human body is fully mastered; and so, also, is microscopic anatomy, as far as the skill of the optician will let it; morbid anatomy has been demonstrated until it has become a nearly wasteful repetition of labor; and surgical art, itself purely experimental, and therefore positive, has progressed so far as to be, in its operative part, all but perfect. Physiology and Pathology, it is true, open before us grand fields of research; but the field of Therapeutics remains comparatively unexplored—and it promises richer fruit, and higher rewards, than any other department of medicine. Empirical observation has done much, but scientific experiment promises far more. If you wish, gentlemen, to become true benefactors to science and humanity, to enrol your names in an honorable niche on the imperishable tablets of our noble art, enter this broad field of experiment and discovery, and contribute your share, however humble, to enlarge the boundaries of Therapeutical Science.

